# Effectiveness of a brief multicomponent intervention to improve physical activity level and functional capacity in fibromyalgia and chronic fatigue syndrome (Synchronize+)

**DOI:** 10.3389/fphys.2024.1441076

**Published:** 2024-12-09

**Authors:** Carme Martín-Borràs, Gemma González Serra, Noèlia Carrasco-Querol, Oriol Sansano-Nadal, Nerea Bueno Hernández, Pau Bestraten del Pino, Marta Pastor Cazalla, Rosa Caballol Angelats, Pilar Montesó-Curto, Elisabet Castro Blanco, Macarena Pozo Ariza, José Fernández-Sáez, M. Rosa Dalmau Llorca, Alessandra Queiroga Gonçalves, Carina Aguilar Martín

**Affiliations:** ^1^ Unitat de Suport a la Recerca Terres de l’Ebre, Fundació Institut Universitari d’Investigació en Atenció Primària Jordi Gol i Gurina (IDIAP JGol), Tortosa, Spain; ^2^ Servei de Rehabilitació d’Atenció Primària Terres de l’Ebre i Hospital Verge de la Cinta Tortosa, Institut Català de la Salut (ICS), Tortosa, Spain; ^3^ Departament d’Activitat Física i Salut, Facultat de Ciències de la Salut (FCS) i Facultat en Psicologia, Ciències de l’Educació i l’Esport (FPCEE) Blanquerna, Universitat Ramon Llull, Barcelona, Spain; ^4^ Departament d’Activitat Física i Fisioteràpia, EUSES Terres de l’Ebre, Universitat Rovira i Virgili, Tortosa, Spain; ^5^ Departament de Medicina i Cirurgia, Programa de doctorat de Biomedicina, Facultat de Medicina i Ciències de la Salut, Universitat Rovira i Virgili (URV), Reus, Spain; ^6^ Fundació Institut Universitari per a la Recerca a l’Atenció Primària de Salut Jordi Gol i Gurina (IDIAPJGol), Barcelona, Spain; ^7^ Servei d’Atenció Primària Terres de l’Ebre, Institut Català de la Salut (ICS), Tortosa, Spain; ^8^ Departament de Medicina i Cirurgia, Facultat de Medicina i Ciències de la Salut, Universitat Rovira i Virgili (URV), Reus, Spain; ^9^ Departament d’Infermeria, Facultat d’Infermeria Campus Terres de l’Ebre, Universitat Rovira i Virgili (URV), Tortosa, Spain; ^10^ Red de Investigación en Cronicidad, Atención Primaria y Promoción de la Salud (RICAPPS), Barcelona, Spain; ^11^ Unitat d’Avaluació i Recerca, Direcció d’Atenció Primària Terres de l’Ebre i Gerència Territorial Terres de l’Ebre, Institut Català de la Salut (ICS), Tortosa, Spain

**Keywords:** fibromyalgia, chronic fatigue syndrome, physical activity, multicomponent intervention, primary health care, muscle strength, cardiorespiratory capacity, functional capacity

## Abstract

**Introduction:**

Fibromyalgia (FM) and chronic fatigue syndrome (CFS) are complex central sensitization syndromes that represent an important public health problem. Low cardiorespiratory fitness and muscle function with habitual intolerance to efforts are common characteristics of FM and CFS. This study aimed to examine the effect of a brief multicomponent intervention based on physical activity (PA), nutrition, and chronobiology on movement behaviors (PA, sedentary and sleep time), muscle strength, and cardiorespiratory capacity.

**Methods:**

randomized controlled trial was conducted in primary healthcare in Catalonia. A total of 143 individuals with FM or FM and CFS concomitantly (age 50.8, SD 8.1; 94.4% women) were randomly allocated to the intervention (IG, n = 69) or control (CG, n = 74) groups. The IG participated in a brief multicomponent (PA, nutrition, and chronobiology) group-based intervention (4 sessions, 3 h/session) while the CG received usual primary care practice. Primary outcome measure was PA measured by the REGICOR-Short Physical Activity Questionnaire. Secondary outcomes were sedentary (International Physical Activity Questionnaire) and sleep time (Pittsburgh Sleep Quality Index), upper- and lower-body muscle strength (handgrip and sit-to-stand test, respectively), and aerobic capacity (6-min walk test). Data were collected at baseline and 3 months post-intervention.

**Results:**

The IG showed positive differences at 3-month follow-up, with highly appreciably PA levels, less sedentary time, and significantly improved sleep time. Significant between-group differences were also observed at 3 months, with better health values in the IG: PA and sleep time (370.3 ± 307.0 vs. 195.9 ± 289.1 min/week and 6.1 ± 1.6 vs. 5.5 ± 1.8 h/night, respectively) and less sedentary time (266.2 ± 153.3 vs. 209.4 ± 199.9 min/day). The IG also showed higher upper limb strength and significant lower-body strength both between and within groups, as well as significantly improved cardiorespiratory capacity.

**Conclusion:**

The Synchronize + multicomponent program implemented at primary healthcare has shown short-term effectiveness in improving 24-h movement behaviors and health outcomes in individuals with FM, with or without CFS. This intervention may be a first step in educating and motivating people with FM and CFS to adopt an active lifestyle, leading to improved health. Long-term follow-up will determine whether the changes are maintained over time and their impact on quality of life and healthcare costs.

## 1 Introduction

Fibromyalgia (FM) and chronic fatigue syndrome (CFS) are often overlapping common central sensitization syndromes with a reported prevalence of 1.0%–2.7% worldwide, affecting especially women (2–3 times higher prevalence than men) (Walitt et al., 2015; Zambolin et al., 2022). Both of these complex, debilitating chronic conditions importantly impact the lives of those diagnosed, causing physical and emotional weakness (Häuser et al., 2015; Rivera et al., 2019). Despite their high prevalence and severity, their etiology and pathophysiology still raise many questions (Antunes and Marques, 2022; Bateman et al., 2021). Inconsistencies in the establishment and application of diagnostic criteria (Lim and Son, 2020; Strand et al., 2019; Zambolin et al., 2022) lead to delayed diagnosis and high rates of underdiagnosis (85%–90%). As a result, patients’ quality of life (QoL) is affected before symptom management can be implemented (Galvez-Sánchez, Duschek, and Del Paso, 2019; Rivera et al., 2019; Zambolin et al., 2022), leading to frustration and distrust of professionals and the healthcare system (Gana, Cuvelier, and Koleck, 2021).

Both diagnoses clearly have a relevant impact on the public health system and society (D’Onghia et al., 2022; Arnold, Gebke, and Choy, 2016). Although these syndromes affect all age groups, they are more prevalent in middle-aged people (FM typically affects people aged 20–50 years, and the average onset age of CFS is 33 years) (Marques et al., 2017; Watson, 2011). This can affect the most productive phase of life (Nacul et al., 2021). Work disability is common in adults with FM and/or CFS (Castro-Marrero et al., 2019; Palstam and Mannerkorpi, 2017). Patients are often unable to work full-time or simply cannot cope with work-related demands (Mannerkorpi and Gard, 2012; National Institute for Health and Care Excellence, 2021). These difficulties frequently result in sick leave and even layoffs, leading to a high unemployment rate in this population (Palstam and Mannerkorpi, 2017; Watson, 2011). To avoid this, some individuals continue to work full-time, sacrificing social and leisure activities in their free time to rest, which affects their overall physical activity (PA) and increases sedentary time (Zambolin et al., 2022).

FM and CFS are characterized by widespread pain, fatigue, sleep disturbances (Abbi and Natelson, 2013; Giorgi et al., 2022), cognitive and psychological symptoms (Antunes et al., 2021; Wasti et al., 2023), reduced cardiorespiratory capacity and muscle function, and intolerance to exertion (Nacul et al., 2021; Nijs et al., 2011; Zambolin et al., 2022). These characteristics affect patients’ physical, psychological, and social health, making it difficult to perform daily activities and fulfill social, family, and work responsibilities (Castro-Marrero et al., 2019; Galvez-Sánchez, Duschek, and Del Paso, 2019; Rivera et al., 2019). FM and CFS also impact daily movement behaviors (PA, sedentary time, and sleep patterns) (Tremblay et al., 2017), leading to decreased PA levels, increased rest time, and poor sleep (Bidonde et al., 2019; Vancampfort et al., 2023). Although FM and CFS may present differently in terms of symptoms and severity, they both have a long-term health impact and are associated with a lower health-related QoL compared to the general population and even to many other chronic or disabling diseases (Lee et al., 2017; National Institute for Health and Care Excellence, 2021).

The evidence confirms the importance and benefits of regular PA, while sedentary time should be reduced (Warburton and Shannon, 2016; Bull et al., 2020). However, only a small portion of the general population follows the World Health Organization (WHO) recommendations and an even smaller percentage of people with FM and CFS lead an ace lifestyle (Segura-Jiménez et al., 2015). Individuals living with FM, with or without CFS, are less physically active and more sedentary than the general population (Nijs et al., 2011; Vancampfort et al., 2023). It is estimated that approximately 73% of FM patients do not act on current recommendations of 150 min of moderate-to-vigorous weekly PA and spend more than 9 h per day being sedentary (Vancampfort et al., 2022), culminating in the known negative health consequences of both behaviors separately (Ekelund et al., 2016; Koster et al., 2012). Regarding sleep, many patients report poor restorative sleep, frequent awakenings during the night, and difficulty falling back to sleep (Kaltsas et al., 2023; Spaeth et al., 2011). Sleep issues may affect other movement behaviors, resulting in an even less active and more sedentary lifestyle (Bidonde et al., 2019; Vancampfort et al., 2023).

There are currently no curative treatments for FM and CFS, nor is there a standardized approach (Häuser et al., 2015). Pharmacological treatments have only shown partial effectiveness (Giorgi et al., 2022). Therefore, recent studies have focused on the effectiveness of non-pharmacological approaches as an alternative and complementary treatment. In this sense, multicomponent group-based interventions are becoming increasingly relevant in the field (Angelats et al., 2019; Carrasco-Querol et al., 2023; Gavilán-Carrera et al., 2023; Macfarlane et al., 2017; Serrat et al., 2020a; Serrat et al., 2020b; Serrat et al., 2021; Häuser et al., 2009). Specifically, physical exercise and education have recently been recommended and applied as a therapeutic, health promotion, and disease prevention tools for FM and CFS (Dures et al., 2023; Thieme et al., 2017; Thompson et al., 2013). Several studies and guidelines on the management of chronic pain and fatigue state that exercise is strongly recommended regardless of what kind (Dures et al., 2023; NICE, 2021). The European Alliance of Associations for Rheumatology (EULAR) also recommends that health professionals refer individuals with inflammatory rheumatic and musculoskeletal disease to PA and/or psychoeducational interventions and encourage them to engage in long-term PA as a lifestyle (Dures et al., 2023).

Recent reviews have concluded that exercise may have specific benefits in reducing the severity of pain and depression, as well as improving functionality in FM patients (Andrade et al., 2020; Geneen et al., 2017). Furthermore, it has been found to have a positive impact on physical and mental health, improving health-related QoL (Couto et al., 2022), and is generally considered safe (Geneen et al., 2017). The same authors report that different types of exercise, including aerobic, resistance, and stretching, have a positive impact on widespread pain, depression, and QoL (Couto et al., 2022). A current meta-analysis indicates that a combination of aerobic and resistance exercise may be the most effective type of exercise for improving QoL, reducing pain, and enhancing physical function in individuals with FM (Vilarino et al., 2022). Despite the demonstrated role of PA in improving health in FM and CFS, there is little evidence regarding the amount of exercise (volume, frequency, and duration) and what kind of intervention design (type of exercise) is effective in improving the main symptoms and lifestyle (Couto et al., 2022). Geneen et al. (2017) reported a wide range of intervention duration (1–30 months), frequency (once weekly to twice daily), session duration (two to 120 min), and intensity (low to moderate-to-high intensity).

Due to the proven role of PA in improving health, particularly in individuals with FM, with or without CFS, interventions are necessary to encourage them in practice. The content and format of these interventions may vary depending on their objectives. However, it is crucial to clarify the prescription of exercise in this population and the context in which it should be prescribed and initiated. Multicomponent interventions that include PA appear to be effective in improving health despite the need for further research. Primary healthcare is an ideal setting for such interventions since they involve different specialists, such as physiotherapists. This study aims to evaluate the effectiveness of the Synchronize + intervention, a short multicomponent group-based intervention, in improving PA levels, strength, and cardiorespiratory capacity among FM and CFS patients.

## 2 Materials and methods

### 2.1 Design

A pragmatic randomized clinical trial (NCT05719493) was conducted as part of the Synchronize + study (Carrasco-Querol et al., 2023) to determine the effectiveness of the Synchronize + intervention. The control group (CG) followed the usual care while the intervention group (IG) also followed a brief multicomponent (interdisciplinary) group-based intervention.

### 2.2 Participants

**FIGURE 1:**
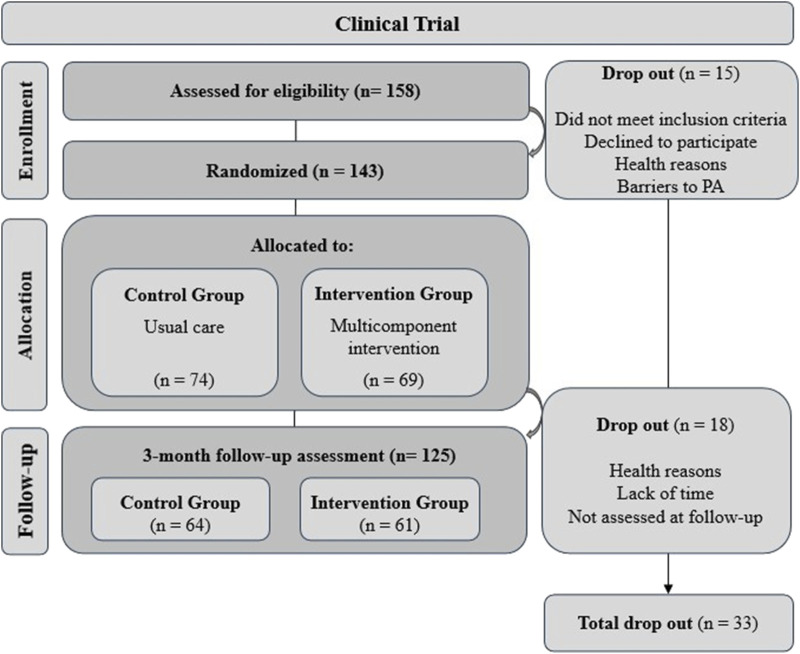
Sample flow chart of the study.

Study participants included people diagnosed with FM, with or without CFS (International Classification of Diseases-10, M79.7, and G93.3, respectively) (WHO, 2019), attended by public primary healthcare centers (PHC) of the Catalan Health Institute (ICS) in the Terres de l’Ebre region (Catalonia). This clinical trial involved a total of 143 patients who were randomly assigned to the IG (n = 69) or CG (n = 74) (Figure 1). Eligibility criteria included people aged 18–65 with a recent diagnosis (<10 years) of FM, or FM and CFS concomitantly, with availability and interest in participating. Individuals were ineligible if they were participating in other ICS group interventions aimed at the treatment of these syndromes, had a diagnosis of severe mental, comorbidity or other relevant medical disorders that may interfere with the development of the intervention. Participation was voluntary. Study information was provided to participants and written informed consent was obtained from all participants before the start of the study. At the first visit, the participants were sequentially allocated to a study group according to the randomization list, following Efron procedure (Efron, 1971) using the IBM SPSS Statistics v.23.0. package for Windows. Participants were informed of which group they were allocated (control or intervention) and the schedule of sessions (or visits) (Carrasco-Querol et al., 2023).

Recruitment process began in October 2021 and continued until total sample was completed. A total of 10 intervention groups were conducted (1rst group: October 2021, last group: May 2023). Before the start of each group, baseline assessments were carried out for all participants. Upon completion of each intervention, planned assessments were conducted (Figure 1).

The Clinical Research Ethics Committee of the Jordi Gol Primary Care Research Institute approved the study (codes: 21/154-P and 22/087-P), which was performed in accordance with the Helsinki/Tokyo Declaration.

### 2.3 Interventions

#### 2.3.1 Synchronize + intervention

Synchronize+ is a brief multicomponent group-based intervention established to actively educate people diagnosed with FM, with or without CFS, on PA, nutrition, and chronobiology. Four group-based sessions were conducted over 2 consecutive weeks for a total of 12 h (2 sessions/week, 3 h/session) at the PHC. General intervention details are available in a previous article (Carrasco-Querol et al., 2023).

**FIGURE 2:**
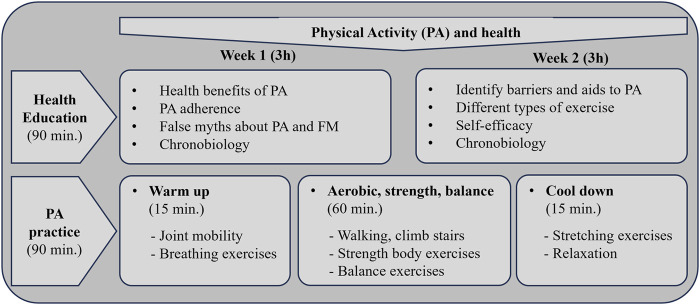
Synchronize+ intervention details on physical activity and health.

PA and health sessions (2 sessions) were divided into health education and PA practice (Figure 2). Health education component included active education on relevant PA content (week 1: benefits, adherence, false myths about PA; week 2: barriers and aids in PA, different types of exercise and self-efficacy; both sessions included chronobiology). Each PA practice session included three parts: first, a warm-up, consisting of walking and full-body mobility exercises, followed by an aerobic activity, strength, and balance exercises, and lastly, a cool-down based on stretching and breathing exercises (see Figure 2). Aerobic activity included basic activities like walking at different speeds and in different ways (e.g., shorter and longer steps). Great emphasis was placed on upper and lower-body strength exercises (e.g., biceps curl, squats, standing leg curl, and especially functional exercises) because most participants had never performed them and did not understand the need to incorporate these exercises regularly.

A previously trained physiotherapist conducted and supervised the PA practice. The practical part of each session aimed to familiarize participants with different types of exercise and at how to perform them safely, with an emphasis on intensity control. Participants were taught to monitor their self-perceived exertion (rate perception exhaustion, RPE) using the Borg scale. Recommended exercise guidelines for people with FM were provided as a reference (Riebe et al., 2018) (Figure 2) to define frequency, intensity, and time. During the sessions, the professional explained the different exercises, demonstrated how they were performed, and then the participants practiced them (2-3 aerobic activities: 4-6 RPE, 4-6 strength exercises per session: 8–12 reps, 2-4 sets per muscle group, 4-6 RPE; 3-5 balance exercises; 4-6 stretching exercises: 10–30 s per stretch). After the practice, the facilitator provided specific, individual corrections for each participant, putting special emphasis on strength exercises. Special emphasis was placed on exercise progression and personalization. During the program sessions, participants were encouraged to include PA in their daily routines, with particular focus on strength exercises. At the end of the intervention, participants were presented options for practicing PA in the local community.

#### 2.3.2 Usual care

The control group followed the usual clinical practice. Usual care usually consists of an individual visit with a FM specialist who inform the patient of the disease using a leaflet. Participants in the control group were also offered the opportunity to participate in the program after the end of the study.

### 2.4 Outcomes and data collection

Sociodemographic (age, gender, educational level, and employment status), clinical (self-reported number of medications and body mass index [BMI]), and comorbidity and illness data (years since FM diagnosis, and presence of CFS) were collected at baseline. BMI categories were stated as underweight and normal weight when BMI was ≤24.9 kg/m^2^, overweight when BMI was 25–29.9 kg/m^2^, and obese when BMI was ≥30 kg/m^2^ (World Health Organization, 1995).

The main outcome measure was PA time (for primary outcome in the overall study see Sinchronize + protocol) (Carrasco-Querol et al., 2023) assessed with the REGICOR-Short Physical Activity Questionnaire (Molina et al., 2017). PA was measured as light (<4 Metabolic Equivalent of Tasks [MET]) or moderate-to-vigorous (4–6 MET) PA intensity and weekly total PA (min/week). Because of the conceptual model of 24-h movement and non-movement behaviors (Tremblay et al., 2017), sedentary behavior and self-reported sleep time were also evaluated as secondary outcomes. The Short-version International Physical Activity Questionnaire (IPAQ) was used to detect sedentary time in a subsample (Meh et al., 2021; Craig et al., 2003), whereas one question (‘How many hours of actual sleep did you get at night?‘) from the Brief version of the Pittsburgh Sleep Quality Index (B-PSQI) (Buysse et al., 1989; Sancho-Domingo et al., 2021) was chosen to assess nightly sleep time.

Secondary outcomes also included cardiorespiratory capacity, assessed using the 6-min walk test (6MWT) (Enright, 2003; Rikli and Jones, 2013), and muscle strength. The sit-to-stand test was used to assess lower-trunk strength, while upper trunk strength was evaluated using the hand grip force test (handgrip GRIPX, Kuptone, EH101) (Rikli and Jones, 2013). Those tests were carried out by a physiotherapist at baseline and 3-months post-intervention.

Data on PA level, sedentary behavior and sleep time, was also collected at baseline and 3-months post-intervention thought online Microsoft forms self-administered questionnaires (REGICOR, IPAQ and B-PSQI).

### 2.5 Statistical analysis

Baseline characteristics were calculated for all variables and presented descriptively as mean and standard deviation (SD) for continuous data, whereas categorical data are shown as absolute number (n) and percentage (%). To examine any possible baseline difference between the CG and IG, Mann Whitney U-tests and the chi-square test were performed. Statistical significance was set at *p* < .05.

Our primary outcome measure was the self-reported leisure-time PA (min/week) and any variation between time spent doing PA at baseline and 3-months follow-up. A Mann Whitney *U* test was performed to assess any difference between groups, as well as for secondary continuous variables. The chi-square test was employed for categorical outcomes to compare differences between the CG and IG. To compare changes over time (baseline vs. 3-months follow-up) within each group, the non-parametric Wilcoxon test was conducted for both primary and secondary outcomes. The effect size to quantify changes between and within groups was also calculated using the means and SD of the groups through Cohen’s d. Results were classified as a small (d > 0.2), moderate (d > 0.5), large (d > 0.8) and very large (d > 1.2) effect (Cohen, 1992). Statistical significance was set at *p* < .05. For the statistical analyses, IBM SPSS Statistics for Windows, version 20.0 (IBM Corp) was used.

An analysis of generalized linear regression modeling was performed to elucidate the impact of the intervention on the primary (physical activity) and secondary outcome measures (sedentary behavior, sleep, hand grip strength, lower-body strength trunk and cardiorespiratory capacity). Generalized linear models (GLMs) are statistical models that permit the modelling of the relationship between a response variable and one or more predictor variables (Zareie et al., 2023). Socio-demographics, clinical data and comorbidity and disease characteristics (presented in Table 1) were introduced as adjustment variables in all models. Statistical significance was set at *p* < .05. To develop the computational models, R 4.4.1. was used.

**TABLE 1:** Baseline characteristics of overall sample and by treatment condition.

	Overall sample (n= 143)	CG (n = 74)	IG (n = 69)	p
Socio-demographics				
Age (years), mean (SD)	50.8 (8.1)	50.9 (8.4)	50.7 (7.8)	0.891
Female, n (%)	135 (94.4)	69 (93.2)	66 (95.6)	0.720
Education level, n (%)				
Basic studies	54 (37.8)	25 (33.8)	29 (42.0)	0.715
Professional development	57 (39.9)	30 (40.5)	27 (39.1)	
Higher studies	17 (11.9)	9 (12.2)	8 (11.6)	
Employment status, n (%)				0.416
Employed	62 (43.4)	33 (45.2)	29 (42.0)	
Non-employed	80 (55.9)	40 (54.8)	40 (58.0)	
Clinical data				
Medications (num), mean (SD)	4.7 (3.3)	4.7 (3.3)	4.8 (3.3)	0.910
BMI categories, n (%)				0.796
Underweight and normal	42 (29.4)	21 (28.4)	21 (30.4)	
Overweight	44 (30.8)	25 (33.8)	19 (27.5)	
Obese	57 (39.9)	28 (37.8)	29 (42.0)	
Comorbidity and illness				
Years since FM diagnosis, n (%)				0.520
Less than 1 year	5 (3.5)	2 (2.7)	3 (4.3)	
Between 1 and 5 years	71 (49.6)	39 (52.7)	32 (46.4)	
More than 5 years	59 (41.3)	27 (36.5)	32 (46.4)	
Unknown	8 (5.6)	6 (8.1)	2 (2.9)	
Chronic fatigue diagnosis, n (%)	80 (55.9)	42 (57.5)	38 (55.1)	0.768
Movement behaviours, mean (SD)				
Physical Activity (min/week)				
Light PA	114.5 (187.5)	116.4 (235.3)	117.2 (130.7)	0.967
Moderate-to-vigorous PA	131.2 (245.6)	145.2 (312.3)	134.4 (177.8)	0.803
Total PA	245.8 (332.7)	261.6 (420.7)	252.2 (238.9)	0.871
Sedentary behaviour^a^ (min/day)	287.9 (148.5)	270.8 (149.8)	315.5 (136.3)	0.206
Sleep (h/night)	5.4 (1.8)	5.3 (1.5)	5.5 (2.0)	0.291
Health outcomes, mean (SD)				
Pain (0–10)	7.26 (1.7)	7.32 (1.6)	7.1 (1.8)	0.376
Muscle strength				
Hand grip strength (kg)	18.35 (9.8)	17.68 (8.2)	19.7 (11.4)	0.223
Lower-body strength, (reps)	7.91 (3.4)	8.16 (3.3)	7.8 (3.4)	0.497
Cardiorespiratory capacity				
6MWT distance (m)	351.4 (86)	344.0 (89.7)	367.5 (77.3)	0.098

IG: intervention group, CG: control group; SD: standard deviation; num: number; BMI: body mass index; FM: fibromyalgia; reps: repetitions; 6MWT: 6-min walk test; RPE: rate perception exhaustion.

^a^ Subsample (n = 67)

## 3 Results

Of the 158 participants assessed for eligibility for the Synchronize + randomized clinical trial, 143 were randomly allocated to either the CG (n = 74) or IG (n = 69) and 125 were assessed at the short-term follow-up. Figure 1 shows a sample flowchart with the number and reasons for dropouts. All participants were diagnosed with FM, with an average of 5.59 years since diagnosis (SD 4.7). The majority were female (94.4%) and also had a diagnosis of CFS (55.9%). The BMI showed a mean value of 28.75 (SD 6.3). No significant differences were found between the IG and CG in terms of sociodemographic, clinical data, comorbidity and illness, movement behaviors (PA, sedentary and sleep time) and health outcomes (lower-trunk strength and cardiorespiratory capacity) at baseline. Table 1 presents descriptive characteristics of the overall sample and by groups.

**FIGURE 3:**
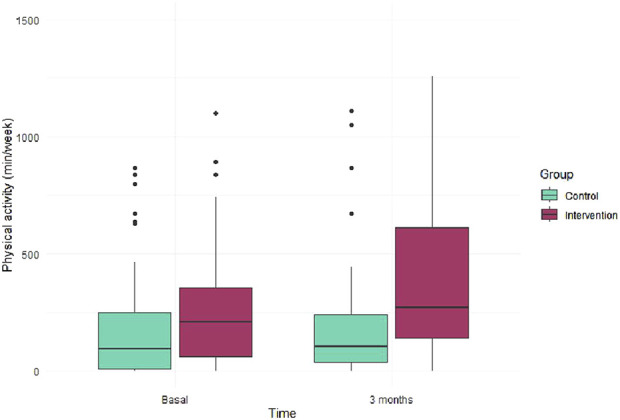
Physical activity time at baseline and 3 month follow-up for control and intervention groups.

Table 2 presents the change of the intervention on movement behaviors and health outcomes by groups. Significant differences were observed in all PA categories (light, moderate-to-vigorous, and total PA time) and sedentary behavior, with higher PA levels and less sitting time in the IG. Figure 3 illustrates that at 3 months, the IG show higher PA values compared to the CG.

**TABLE 2:** Effects of the intervention by group for moviment behaviours and health outcomes.

	Control group (CG) Mean (SD)					Intervention group (IG) Mean (SD)				
	Baseline	3 months follow-up	Δ	95%IC	p	Baseline	3 months follow-up	Δ	95%IC	p
Movement behaviours^a^										
PA (min/week)										
Light PA	116.4 (235.3)	78.5 (112.8)	−32.5	(−37.5 to −18.6)	0.213	117.7 (130.7)	162.1 (158.0)	37.7	(35.8–40.9)	**0.013**
Moderate-to-vigorous PA	145.2 (312.3)	117.4 (227.7)	−19.2	(−19.9 to −17.0)	0.141	134.4 (177.8)	208.2 (207.1)	54.9	(47.5–69.2)	**<0.001**
Total PA	261.6 (420.7)	195.9 (289.1)	−25.1	(−25.3 to −24.6)	0.059	252.2 (238.9)	370.3 (306.9)	46.8	(45.0–49.7)	**<0.001**
SB^b^ (min/day)	270.8 (149.8)	209.4 (199.9)	−22.7	(−8.4 to −43.4)	0.165	315.5 (136.3)	266.2 (153.3)	−15.6	(−30.5 to −4.8)	0.937
Sleep (h/night)	5.3 (1.5)	5.5 (1.8)	2.6	(0.8–3.9)	0.495	5.6 (2.0)	6.1 (1.6)	8.0	(6.2–10.2)	**0.003**
Health outcomes										
Strength										
Hand grip strength (kg)	17.7 (8.2)	18.2 (8.9)	3.4	(2.0–4.5)	0.258	19.7 (11.4)	20.88 (9.5)	5.9	(3.9–8.7)	0.065
Lower body strength (reps)	8.2 (3.3)	8.4 (3.5)	3.4	(2.4–4.0)	0.722	7.9 (3.4)	9.63 (3.2)	23.8	(21.9–26.3)	**<0.001**
Cardiorespiratory capacity										
6MWT distance (m)	344.0 (89.7)	360.8 (80.8)	4.9	(5.6–4.3)	0.192	367.5 (77.3)	402.2 (76.6)	9.4	(9.3–9.6)	**<0.001**
RPE before 6MWT (0–10)	2.7 (1.7)	2.7 (1.9)	2.3	(0–3.9)	0.575	2.7 (1.8)	1.9 (1.8)	−30.4	(−38.0 to −24.8)	**0.025**
RPE after 6MWT (0–10)	4.7 (1.9)	4.5 (2.0)	−4.5	(−3.1 to −5.9)	0.978	4.9 (2.3)	3.4 (2.3)	−30.9	(−35.8 to −27.2)	**0.002**

SD: standard deviation; IC: interval confidence; Δ: increment; PA: physical activity; SB: sedentary behavior; reps: repetitions; 6MWT: 6-min walk test; RPE: rate perception exhaustion.

^a^ Self-reported time;

^b^ Subsample (n = 67). The number of participants varied across assessments (see Figure 1). Bold fonts stand for different variables and for significant values.

Additionally, the IG demonstrated significant lower-body strength and better cardiorespiratory capacity (meters walked and perceived exertion measured before and after performing the 6MWT). Although trends towards improvement were noted in sedentary time and hand grip strength, these results were not statistically significant. Between-group significant differences were showed for the same outcomes (see Table 3). When analyzing the effect size of the intervention using Cohen’s d, changes in the IG showed a moderate effect size on light PA, total PA, and 6MWT distance (d > 0.50). The effect size was smaller for the remaining changes observed between groups at 3-months follow-up.

**TABLE 3:** Effects of the intervention between groups for moviment behaviours and health outcomes.

	CG vs. IG (3 months follow-up)			
	Δ	95%IC	d	p
Movement behaviours^a^				
Physical Activity (min/week)				
Light PA	106.3	(89.7–141.4)	*0.608	**0.001**
Moderate-to-vigorous PA	77.4	(49.9–157.0)	0.417	**<0.001**
Total PA	89.0	(67.4–135.8)	*0.585	**<0.001**
Sedentary behavior^b^ (min/day)	27.1	(18.4–47.6)	0.319	0.162
Sleep (h/night)	11.5	(10.0–13.8)	0.376	**0.032**
*Health outcomes*				
Strength				
Hand grip strength (kg)	14.2	(13.9–14.7)	0.283	0.092
Lower body strength trunk (reps)	14.1	(12.7–16.1)	0.354	**0.040**
Cardiorespiratory capacity				
6MWT distance (m)	11.5	(10.8–12.1)	*0.525	**0.001**
RPE before 6MWT (0–10)	−30.9	(−38.0 to −26.0)	0.449	**0.014**
RPE after 6MWT (0–10)	−23.5	(−28.79 to −19.28)	0.491	**0.008**

CG: control group; IG: intervention group; IC: interval confidence; Δ: increment; d (Cohen’s d): effect size (*>0.50 moderate; **>0.80 large; ***>1.2 very large); 6MWT: 6-min walk test; RPE: rate perception exhaustion.

^a^ Self-reported time;

^b^ Subsample (n = 67). The number of participants varied across assessments (see Figure 1). Bold fonts stand for different variables and for significant values

The generalized linear model applied to PA, sedentary time, sleep, strength and 6MWT distance indicates that the intervention significantly influenced changes in physical activity and cardiorespiratory capacity. Age played a significant role in the values obtained for strength and 6MWT distance (see Table 4).

**TABLE 4:** Estimated association between outcome variables and intervention participation adjusted for socio-demographics, clinical data, comorbidity and illness characteristics using GLM.

	PA total time	Sedentary time	Sleep	Handgrip strength	Lower-body strength	6MWT distance
Intercept						
Estimate (ß)	725.0	259.2	7.3	60.5	15.0	476.6
%95 IC	64.9–1,385.1	−641.1 to 1,159.5	3.6–10.9	45.0–76.1	8.5–21.6	337.5–615.6
p	**0.034**	0.579	**<0.001**	**<0.001**	**<0.001**	**<0.001**
Intervention						
Estimate (ß)	162.0	−23.1	0.6	2.2	1.0	39.5
%95 IC	41.6–282.3	−198.1–151.9	0–1.3	−0.6–5.0	−1.9–2.2	14.7–65.2
p	**0.009**	0.798	0.060	0.127	0.102	**0.002**
Age						
Estimate (ß)	−2.6	4.7	0	−0.2	−0.1	−1.8
%95 IC	−11.3–6.1	−5.9–15.4	−0.1–0	−0.4–0.01	−0.2–0	−3.6–0.1
p	0.559	0.396	0.437	**0.046**	**0.039**	**0.045**
**G**ender (female)						
Estimate (ß)	−86.2	3.1	0.5	−18.5	1.1	51.5
%95 IC	347.9–175.6	−261.3–255.0	−1.0–2.1	−24.7–−12.4	−3.7–1.5	−106.0–3.0
P	0.520	0.981	0.502	**<0.001**	0.407	0.067
Education level (HS)						
Estimate (ß)	76.0	331.2	0.5	−2.4	2.0	127.8
%95 IC	−291.3–443.3	−170.1–832.5	−1.5–2.5	−11.0–6.3	−1.6–5.6	51.0–204.5
p	0.686	0.209	0.624	0.591	0.285	**0.001**
Employment (E)						
Estimate (ß)	−26.2	−5.2	−0.7	0.8	−0.1	25.6
%95 IC	167.2–114.7	−164.4–154.1	−1.4–0.1	−2.5–4.1	−1.5–1.3	−3.9–55.2
P	0.716	0.950	0.090	0.635	0.848	0.092
Medications						
Estimate (ß)	−4.7	17.5	−1.0	0	−0.03	−2.2
%95 IC	−27.5–18.0	−12.0–46.9	−0.2–0	−0.6–0.5	−0.3–0.2	−6.9–2.6
p	0.684	0.258	0.137	0.883	0.768	0.372
BMI						
Estimate (ß)	−2.6	−4.1	0	0	0	−0.1
%95 IC	13.2–7.9	−16.5–8.2	0–0.1	−0.3–0.2	−0.1–0.1	−2.3–2.1
P	0.625	0.517	0.868	0.855	0.380	0.912
Years since FM						
Estimate (ß)	−1.9	−6.2	0	0.2	0.1	−0.3
%95 IC	−17.4–13.5	−33.0–20.6	0–0.1	−0.1–0.6	−0.1–0.2	−3.5–2.9
p	0.806	0.655	0.848	0.223	0.318	0.837
CFS diagnosis						
Estimate (ß)	−47.9	5.1	0	−2.2	0.7	−3.4
%95 IC	−178.1–82.2	−186.4–196.6	−0.8–0.7	−5.3–0.8	−0.5–2.0	−30.7–23.9
p	0.472	0.959	0.920	0.157	0.255	0.807
Pain (0–10)						
Estimate (ß)	−21.9	−47.3	−0.1	−1.9	−0.6	−10.6
%95 IC	−55.7–11.9	−99.1–4.5	−0.3–0.1	−2.7 to −1.1	−0.9 to −0.2	−17.7 to −3.6
P	0.207	0.088	0.452	**<0.001**	**0.001**	**0.004**

GLM: generalized linear model; 6MWT: 6-min walk test; IC: interval confidence; HE: higher education; E: employed; BMI: body mass index; FM: fibromyalgia, CFS: chronic fatigue syndrome.

## 4 Discussion

The purpose of this study was to provide results on the short-term effectiveness of the Synchronize + multicomponent group-based intervention to enhance physical activity levels in FM, with or without CFS. The main findings of this study were: (1) a brief multicomponent intervention based on PA, nutrition and, chronobiology was effective in increasing PA levels among FM individuals at 3-months post-intervention; and (2) the program led to improvements in sedentary and sleep time, lower- and upper-body strength, and aerobic capacity.

International treatment recommendations highlight the importance of multicomponent therapies that include PA and education in improving health outcomes and reversing the vicious cycle of pain, reduced PA, sedentary behavior, and disability (Borisovskaya et al., 2020). Likewise, EULAR suggests that this is a first step to becoming aware of the importance of PA, in closely linking it with patient education (Dures et al., 2023; Thieme et al., 2017). Our results are consistent with previous literature on multicomponent treatment interventions for FM and show that an approach based on educational and PA components is effective in improving movement behaviors and physical function in FM (Serrat et al., 2021; Serrat et al., 2020a; Serrat et al., 2020b). In this sense, Synchronize + offers a brief, personalized, and supervised group-based intervention to motivate, initiate, and enhance self-efficacy in PA and to educate patients on an active and healthy lifestyle.

When analyzing movement and non-movement behaviors in 1 day, the Synchronize + IG showed healthier values in terms of PA, and sedentary and sleep time. However, the overall sample (including IG and CG) did not report any self-reported behavior for a large part of the day (24 h), suggesting a possible underestimation in the various movement behaviors. The Synchronize + study used the self-report method to asses movement behaviors due to its practicality, low cost, and general acceptance (Dishman et al., 2001), but self-reporting may lead to over- or underestimates of true PA and rates of inactivity (Prince et al., 2008). In fact, people tends to overestimate PA while underestimating sedentary behavior is common due to unawareness of time spent sitting (Sansano-Nadal et al., 2022). When PA was assessed, contrary to expectations, participants underestimated PA levels, which could be explained by focusing on leisure-time PA and not taking into account the broader concept of PA, which includes work, transport and domestic activities (Quinn and Barone Gibbs, 2023). In any case, the increase in PA levels (especially moderate-to-vigorous PA), the reduction in sedentary time, and the increase in sleep time are all positive outcomes.

As far as we know, there are no brief multicomponent interventions that have evaluated different movement behaviors according to Tremblay et al.‘s conceptual model (Tremblay et al., 2017). This is relevant due to its relationship with several health outcomes (Janssen et al., 2020). Recently, the Canadian Society for Exercise Physiology published guidelines that underscore the importance of movement behaviors throughout the 24-h day, suggesting that adults: (1) should be physically active every day (several hours of light PA, including standing); (2) should minimize sedentary time (8 h or less, no more than 3 h of recreational screen time and break up long periods of sitting); and (3) should get enough sleep (between 7 and 9 h of good quality sleep) for health benefits (Ross et al., 2020).

When assessing the PA effects of the Synchronize + intervention independently, significant increases in light PA and moderate-to-vigorous PA were observed, indicating a healthy change in movement patterns. This is particularly relevant for individuals with FM, with or without a diagnosis of CFS, who tend to spend less time engaging in PA and more time sitting compared to individuals of the same age and sex without these diagnoses (Nijs et al., 2011; Vancampfort et al., 2022; Vancampfort et al., 2023). When compared to other FM interventions that include PA, Synchronize + participants have lower levels of PA and spent less time on sedentary behavior at both baseline and short-term follow-up. This may be attributed not only to using a self-reporting instrument but also to the different questionnaires used in each study. The REGICOR-Short Physical Activity Questionnaire inquires about monthly frequency of six activities: walking, brisk walking, gardening, walking trails, climbing stairs, and sports activities (Molina et al., 2017). This poses a major recall challenge, as it is easy to report only scheduled activities without considering many minutes of daily PA.

As for sedentary behavior, healthcare professionals should inform FM patients that reducing sedentary time may be a relevant strategy to manage symptoms, including widespread pain. A recent study suggests that replacing 30 min of sedentary time with light PA in FM patients was associated with reduced body pain, lower disease impact, more vitality, and better social and physical function (Vancampfort et al., 2023). The IPAQ assessment of sedentary time in the IG yielded lower results than expected. Moreover, there is a well-known tendency of self-reported tools to underestimate sitting time (Sansano-Nadal et al., 2022), and the IPAQ sedentary question was non-specific (Meh et al., 2021; Craig et al., 2003). To gain a better understanding of our findings, it may be useful to include more specific questionnaires and objective measures. Finally, the improvement in the amount of sleep time is very similar to the recommendations in current guidelines (Ross et al., 2020).

The evidence shows a significant relationship between physical function and movement behaviors (Gavilán-Carrera et al., 2020). Muscle strength and cardiorespiratory endurance are common indicators of health and functional capacity, which are determinants of independence in performing daily activities (den Ouden et al., 2013). Individuals with FM and CFS show lower values of both functional components, and usually report discomfort during exercise and reduced exercise tolerance (Nacul et al., 2021; Nijs et al., 2011; Zambolin et al., 2022). Recent reviews have concluded that exercise has many health benefits for FM and other chronic conditions and helps improve functionality (Geneen et al., 2017; O’Dwyer et al., 2019). The Synchronize + intervention led to improvements in physical health components. This is consistent with the findings of several systematic reviews and meta-analyses, which have shown that supervised aerobic and resistance training programs significantly improve physical and psychological function in women with FM (Masquelier and D’haeyere 2021). While traditional exercise interventions have focused on aerobic exercises, the Synchronize + program incorporates strength exercises and education on the importance of include it in daily time. Strength training has proven essential in managing various chronic conditions associated with pain, being beneficial to treat FM. However, there is enough evidence on effective protocols to improve symptoms (Andrade et al., 2018). The emphasis on strength in the Synchronize + intervention has led to improvements in cardiorespiratory capacity as observed in the 6-minute-walk test and perceived exertion during the test. Worse QoL is associated with patients with higher levels of sedentary behavior, especially in terms of physical function (Gavilán-Carrera et al., 2020), so this may be another reason for the better physical function shown by the IG in our study.

The Synchronize + intervention was designed based on collaborative design strategies, with the participation of patients with FM and CFS and PA specialists. It should be noted that it is a brief, primary healthcare, group-based, multicomponent (interdisciplinary) intervention. A short intervention is easy to implement in clinical practice as well as a compatible tool with daily family and work routines. Furthermore, it facilitate specific logistic barriers such as time, which may interfere with treatment adherence. Primary healthcare plays a very important role in educating and empowering patients to improve their health and is an optimal setting to enhance PA (Martín-Borràs et al., 2018). Although PA programs are increasingly prevalent in healthcare systems for a variety of chronic pain conditions, few interventions are implemented in primary care for people with FM, with or without CFS. These two diagnoses often overlap and are characterized by persistent fatigue and low exercise tolerance (Nacul et al., 2021; Nijs et al., 2011; Zambolin et al., 2022). These symptoms can be an initial barrier to initiating regular PA. Therefore, it is important to establish health programs that motivate FM patients to improve their active lifestyle. Furthermore, there is evidence of the effectiveness of interventions in the healthcare setting for this group (Giné-Garriga et al., 2013; Martín-Borràs et al., 2018).

Interdisciplinary interventions that include education and prioritize supervised PA have shown greater effectiveness than pharmacological treatment and have been found to be cost effective in managing the chronic primary pain population (NICE, 2021). Additionally, there is evidence supporting the effectiveness of interventions in the healthcare setting to modify behaviors such as increasing PA and reducing sedentary time (Giné-Garriga et al., 2013; Martín-Borràs et al., 2018). The fatigue and low exertion tolerance commonly experienced by individuals with FM and CFS can prevent them from starting regular PA. Individuals with FM often avoid physical exercise because they believe it may aggravate their symptoms, tending to be more sedentary (McBeth et al., 2010). Therefore, it is crucial to establish health programs that motivate FM patients to lead a more active lifestyle by overcoming existing barriers and to offer a positive exercise experience to promote adherence to physical exercise in the future (Rogerson et al., 2016). The inclusion of PA professionals and nutrition specialists in primary care is essential for the successful implementation of such programs. Physiotherapists are trained to promote safety and healthy PA strategies for FM and other chronic conditions and diagnoses (Larsson et al., 2020). Although PA programs are increasingly prevalent in healthcare systems for a variety of chronic pain conditions, few interventions have been implemented in Catalan primary care for people with FM, with or without CFS.

## 5 Limitations

Some limitations of the present study are worth noting. The use of self-reported measures to assess PA levels, and sedentary and sleep time should be considered a limitation due to the risk of bias. The use of objective measures to assess movement and non-movement behavior should be considered for further studies. In addition, sedentary time was assessed using the IPAQ in a sub-sample through a quite general question about sedentarism (Meh et al., 2021). More specific questionnaires should be used to understand daily behaviors besides objective measures. Similarly, the same limitation applies to PA levels. There may also be greater variability in how people with FM self-report than in healthy controls (McLoughlin et al., 2011). Therefore, measurement of PA and also sedentary time in FM individuals should not be limited to self-report measures.

## 6 Conclusion

The Synchronize + intervention significantly improved the PA levels, sedentary and sleep time, muscle strength, and cardiorespiratory capacity of persons with FM and/or CFS at 3-months post-intervention. Further evaluation of long-term adherence and associated health benefits is needed. Synchronize+ is an intervention with interesting potential in chronic disease management and health promotion.

Multicomponent group-based interventions show promise as a safe and effective approach for managing FM and CFS. Future research should focus on standardizing intervention protocols, incorporating long-term follow-up assessments, and exploring individual variations in treatment response. This will help establish clearer guidelines for the implementation of group-based interventions as part of a comprehensive management strategy for FM and CFS. Ultimately, addressing the complex needs of individuals with these conditions requires a multifaceted approach, and group-based interventions hold great potential in this regard.

## Data Availability

The raw data supporting the conclusions of this article will be made available by the authors, without undue reservation.
